# Mimicking the Nitric Oxide‐Releasing and Glycocalyx Functions of Endothelium on Vascular Stent Surfaces

**DOI:** 10.1002/advs.202101788

**Published:** 2021-07-07

**Authors:** Nan Lyu, Zeyu Du, Hua Qiu, Peng Gao, Qin Yao, Kaiqin Xiong, Qiufen Tu, Xiangyang Li, Binghai Chen, Miao Wang, Guoqing Pan, Nan Huang, Zhilu Yang

*Adv. Sci*. **2020**, *7*, 2002330

DOI: 10.1002/advs.202002330

**Figure 4:**
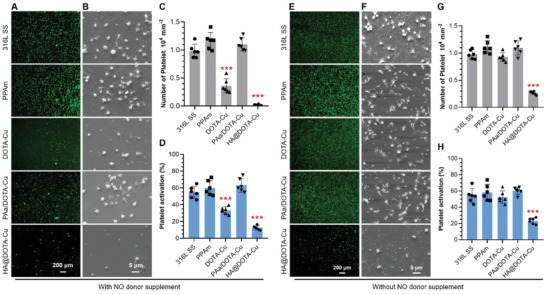
Fluorescence A) and E) and SEM B) and F) images of platelet adhesion on 316L SS surface, PPAm, DOTA‐Cu, PAa/DOTA‐Cuand HA@DOTA‐Cusurfaces with or without NO donor (10 µM GSNO and 10 µM GSH). Theamount of adherent platelets C) and G) and activated platelets D) and H) were obtained by counting and the GMP‐140 assay. Data presented as mean ± SD and analyzed using a one‐way ANOVA, **p < 0 0.01, ***p < 0 0.001.

**Figure 7:**
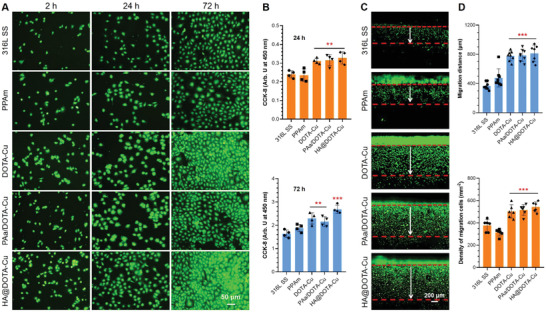
A) Fluorescence staining of human umbilical vein endothelial cells (HUVECs) on different surfaces after culture for 2, 24 and 72 h with NO donor (10 µm GSNO and 10 µm GSH). B) The proliferation of HUVECs in culture media with donor for 24 and 72 h. C,D) Migration of HUASMCs on different surfaces after 1 day of culture with donor supplement. Data are presented as the mean ± SD (*n* = 4) and analyzed using one‐way ANOVA, *p < 0.05, **p < 0.01, ***p < 0.001.

**Figure S6:**
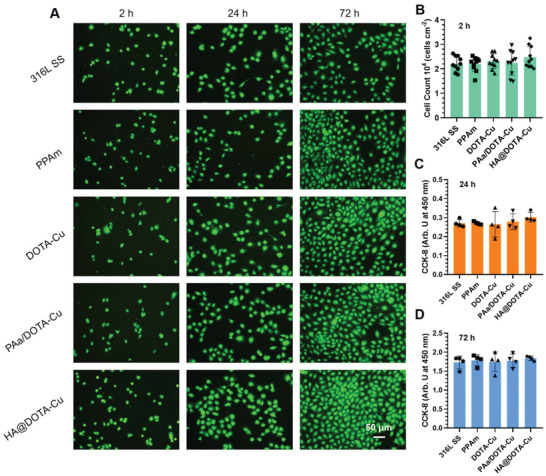
A) Fluorescence staining of HUVECs on the samples surfaces after culture for 2, 24 and 72 h without NO donor. B) Cell count (calculated from at least 12 images), and proliferation of HUVECs cultured in cell media with NO donor for 24 and 72 h C). Data are presented as the mean ± SD (n = 4) and analyzed using one‐way ANOVA, *p < 0.05, **p < 0.01, ***p < 0.001.

In the originally published article, Figure 4b, 7a and S6 (Supporting Information) were wrong. Please find the correct Figure 7 and Figure S6 here:

The authors apologize for any inconvenience this may have caused.

